# Resilience in Pre-Columbian Caribbean House-Building: Dialogue Between Archaeology and Humanitarian Shelter

**DOI:** 10.1007/s10745-015-9741-5

**Published:** 2015-04-23

**Authors:** A. V. M. Samson, C. A. Crawford, M. L. P. Hoogland, C. L. Hofman

**Affiliations:** University of Cambridge, Cambridge, Cambridgeshire UK; Centre for Urban Sustainability and Resilience, Department of Civil Engineering and Geomatic Engineering, University College London, London, UK; Faculty of Archaeology, Leiden University, Leiden, The Netherlands

**Keywords:** Pre-Columbian Caribbean, Resilience, House architecture, Humanitarian shelter, Environmental hazards

## Abstract

This paper responds to questions posed by archaeologists and engineers in the humanitarian sector about relationships between shelter, disasters and resilience. Enabled by an increase in horizontal excavations combined with high-resolution settlement data from excavations in the Dominican Republic, the paper presents a synthesis of Caribbean house data spanning a millennium (1400 BP- 450 BP). An analysis of architectural traits identify the house as an institution that constitutes and catalyses change in an emergent and resilient pathway. The “Caribbean architectural mode” emerged in a period of demographic expansion and cultural transition, was geographically widespread, different from earlier and mainland traditions and endured the hazards of island and coastal ecologies. We use archaeological analysis at the house level to consider the historical, ecological and regional dimensions of resilience in humanitarian action

## Introduction

Archaeologists and international humanitarian organisations are both involved in recovery: The former do this for the past, and the latter for the present. This paper suggests that examination of patterns in the regional archaeological record may reveal some useful implications for dealing with post-disaster shelter relief in the Caribbean and further afield.

The archaeological analysis offered here identifies shared domestic building practices that spanned the islands of the Caribbean archipelago and coalesced into a specific architectural mode. By analysing pre-Columbian house structures from the perspective of environmental and hazard response and distinguishing island house features from those of the mainland, we show the specific ways in which climate change, perceptions of risk, and weather regimes are incorporated within the structure of the house. The material evidence shows the ways in which indigenous Caribbean societies developed house- building practices suited to maritime ecologies and hazards, and which endured over long periods of time. An archaeological perspective on long-range regional change could enhance humanitarian efforts to respond to contemporary physical hazards. In turn, of course, an awareness of humanitarian approaches to alleviating disasters emphasises the agency and hazard resilience of households and enriches archaeological analysis.

Charles Redman (2012), drawing on the work of geographers and resilience theorists, identifies building technology as an important and perennial domain of problem-solving strategies that people use to cope with challenges to their social and physical environment. Elsewhere, in addressing resilience Redman and Kinzig (2003) highlight how institutions that mediate human-environmental interactions emerge, what influences their character, and their effectiveness in mitigating the consequences of natural or man-made disasters. We follow these approaches in examining houses and households as institutions at the frontline of resilience strategies.

Turning briefly to international humanitarian disaster relief it seems that “--providing adequate shelter is one of the most intractable problems in international humanitarian response” (HERR 2011). A primary problem is the tendency to focus narrowly on supplying shelter-related products and replacing physical assets (Bhattacharjee *et al*. 2007; Da Silva 2010:3) rather than on supporting people in rebuilding with locally appropriate building practices (Langenbach 2009; Langenbach *et al*. 2010; Langenbach *et al*. 2006). A second problem is the formal separation - through funding streams and organisational structures - of humanitarian activities into sectors (Pain and Levine 2012), which disconnects shelter and housing from other fields of action affecting the household (Crawford 2011).

**Fig. 1:**
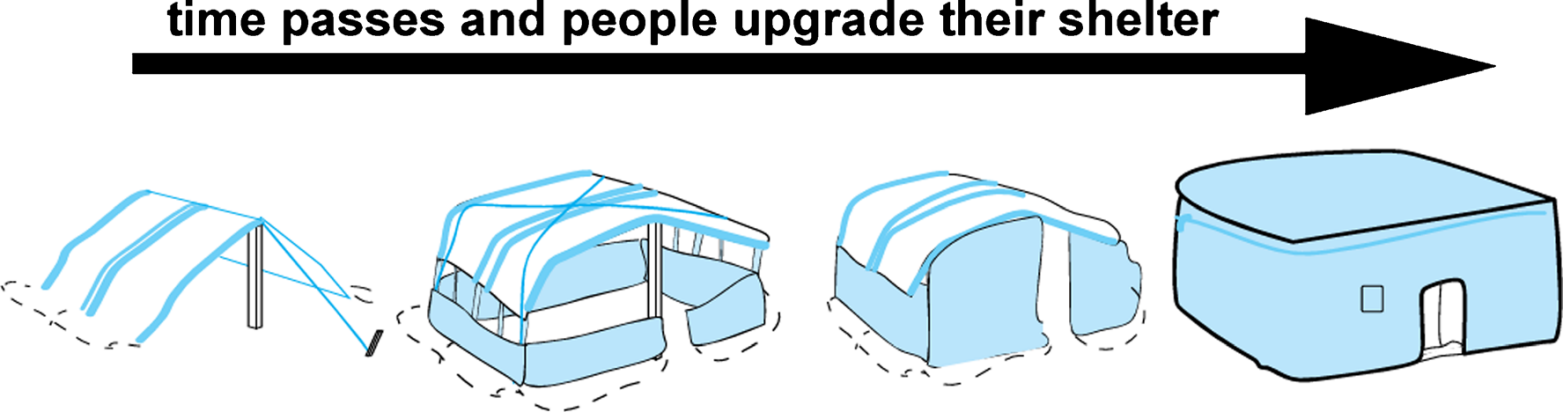
Illustration of the humanitarian shelter process showing incremental upgrades to individual post-disaster shelters (based on UNOCHA 2004)

Similarly, in terms of timeframes, international responses separate funding streams and participant organisations into two distinct phases: emergency response and longer term recovery development (Pain and Levine 2012). This, combined with an immediate, singular focus on the area directly affected by the current disaster, contributes to the tendency to overlook longer term or systemic issues (Burnell and Sanderson 2011; Clermont *et al*. 2011; Crawford 2011) as well as the broader environmental context (Davis 2011). The International Federation of the Red Cross (IFRC), an entity charged with coordinating shelter relief after natural disasters, describes recovery as including“--a process of ‘sheltering’ done by affected households with different materials, technical, financial and social assistance…”.^1^ However, the literature on disaster response suggests that in reality the humanitarian relief often fails to relate to practicalities of livelihood and social and economic life (Davis 2011; Lyons *et al*. 2011; Schilderman and Lyons 2011). The structural separation of sectors and timeframes is exacerbated by conceptual simplifications of the shelter recovery pathway. Figure 1 shows this as a series of incremental improvements that happen only to the *post*-disaster ‘shelter object’ on a single trajectory. This largely ignores the pre-disaster role of people as the agents of change or stasis and assumes a theory of change that depends on a single forward trajectory based on incremental change to the shelter object, ignoring prior, inexplicable or cyclic changes that are in train before and continue after humanitarian history begins at the moment of disaster (Crawford *et al*. 2013).

The assertion that “shelter must be considered as a process not as an object” is not new (Davis 1978: 6), but the romantic appeal of vernacular architecture can mask the messy, ‘unsentimental resourcefulness’ of ordinary building (Richards 2012). Humanitarian narratives of livelihoods and operational standards tend to the normative and prescriptive, giving preeminence to the ‘existing’, ‘appropriate’ and ‘local’ (Sphere 2004) without curiosity as to how these have come about (TEC 2006).

Archaeological perspectives indicate that “…More frequent and more sudden hazards are often prominently encoded in local knowledge“ (Redman 2012:238) and offer a method for viewing this on a broad geographic scale and for a significant period of time (Cooper 2012).

## Historical Maritime Ecologies and Caribbean Domestic Architecture

The diversity of island landscapes across the Caribbean archipelago is unified by a shared maritime climate, diurnal variation in wind and temperature, and periodic dramatic climatic and seismic events. Hurricanes, tropical storms, tsunamis and to a less frequent extent earthquakes and volcanoes are a regular experience of Caribbean peoples past and present (Bérard *et al*. 2001; Delpuech 2004; Hofman and Hoogland 2015; Fitzpatrick 2011; Petitjean Roget 2001; Pielke *et al*. 2003; Roobol and Smith 2004; Scheffers *et al*. 2005). From a long-term perspective, paleoclimatic records show that there were significant changes in climatic conditions throughout the Late Holocene in which centuries-long cycles of wetter, stormy conditions were interspersed with drier conditions and sea-level rise (Beets *et al*. 2006; Cooper 2013; Hodell and Curtis 1991; Donnelly and Woodruff 2007; Malaizé *et al*. 2011). Archaeological research has shown climatic variation had a significant local impact on settlements which led to site abandonment and repeated flooding (Hofman and Hoogland 2015; Fitzpatrick 2011; Rodríguez Ramos 2010:188).

From the time of initial human colonisation of the archipelago ca. 7500BP, peoples in the islands adapted to maritime ecosystems and weather regimes markedly different from the continent of South America. Human habitation and exploitation of island environments impacted local ecosystems, including direct and indirect introduction of exotic plants and animals and forest clearance creating increasingly open, agricultural landscapes, intensifying from 2500BP. Caribbean peoples relied on a diversity of marine, wild and cultivated foods and settled the majority of the Antillean archipelago, including small islands (Boomert 1999; Fitzpatrick and Keegan 2007; Hofman 2013; Keegan *et al*. 2008; Veloz Maggiolo 1991). Caribbean communities were involved in intensive local and regional networks of human mobility and exchange of goods and ideas since first colonisation of the islands (Hofman and Bright eds 2010).

## Regional Narratives: Transformation of the Household Driving Regional Social and Political Change

From 1400BP/AD600 changes in material culture, and the organisation of the landscape, which included the development of settlement hierarchies and ceremonial centres in the Greater Antilles and Virgin islands, have been interpreted as social and political transformations as a result of migrations, the emergence of new and local networks, and environmental change. These changes, which mark the transition to the period known as the Late Ceramic Age, also coincide with the moment in which indigenous house plans first become archaeologically visible. Transformations at the household level such as a proposed shift from communal to lineage-based society in the Greater Antilles, changes in land tenure and heritable property, and a greater investment in place were apparently some of the driving forces behind these higher level changes (Curet and Oliver 1998; Torres 2012; Veloz Maggiolo 1984).

## Archaeological Context

Data needed to make regional or even intra-site comparisons of Caribbean domestic structures were lacking until the last decade. A synthesis of data from excavated structures suggests it is within the post-1400BP cultural and ecological context that we first recognise the emergence of a “Caribbean architectural mode.” The perishable nature of indigenous construction materials and the vulnerability of cultural heritage in the Caribbean complicate the recovery of settlement features (Curet 1992:161; Siegel and Righter 2011; Hofman *et al*. 2012). Instead, information on domestic life and house structures has been inferred from areas with different, continental ecologies and below the hurricane boundary, or from European descriptions in colonial texts. Sixteenth-century sketches and recollections of house structures on Hispaniola (Haiti and the Dominican Republic) the Bahamas, and Cuba (Fernández de Oviedo y Valdéz 1851; Las Casas 1875) describe houses resembling straw huts or tents, with status differences marked by house size, and irregularly laid-out in settlements consisting of one to several thousand structures. These sources refer to a short window following European discovery and conquest in which descriptions unfavourably ranked native technologies against supposedly superior European design. The colonizer’s perspective emphasized expediency and insubstantiality, which, as we shall see, does not correspond to the long-term, material evidence from archaeology.

Archaeologists supplemented this picture with ethnographic data from lowland South America. One model commonly used as a template for ancient or pre-1400BP Caribbean houses is the maloca, or community house, a large, single-dwelling that housed the whole village, up to 40 m in diameter and 30 m in height, often with fully closed walls. This was formerly the dominant type of settlement in Amazonia and the Guianas (Boomert 2000: 283; Versteeg and Schinkel 1992). Other types of traditional tropical settlement include individual family houses around a central clearing, and linear villages along river channels (Boomert 2000; Heckenberger and Petersen 1995; Schinkel 1992; Siegel 1992, 1996).

### Recent Archaeological Excavations

**Fig. 2:**
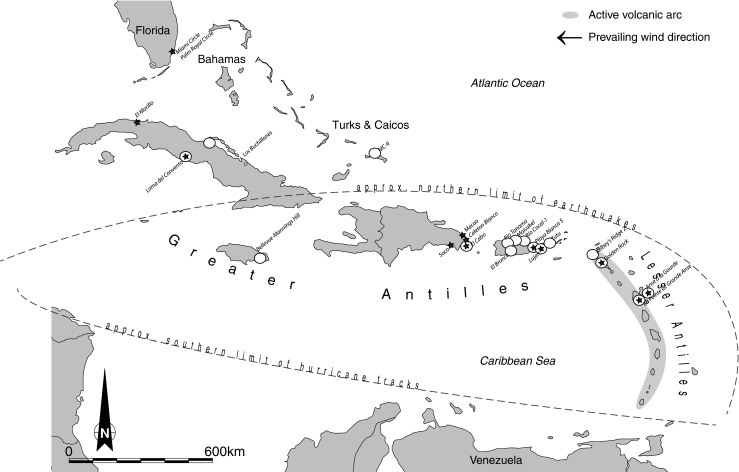
Map of Caribbean with sites in Tables 1 and 2 and environmental characteristics. Sites with complete archaeological plans (*white dots*) and use of bedrock for structure foundations (*black stars*)

A review of more recent archaeological excavations reveals a substantial dataset of complete domestic structures. Most have been recovered from the period post-1100BP from the larger Caribbean landmasses, especially Puerto Rico, with others on Cuba, the Turks and Caicos Islands, Jamaica and St Thomas, U.S. Virgin Islands, and fewer from the Lesser Antilles.^2^ This paper concentrates on sites with published structure plans from the Greater Antilles, Turks and Caicos, Virgin Islands, and northern Lesser Antilles (Fig. 2). We exclude the southern Lesser Antilles due to current data limitations (but see Hofman and Hoogland 2012), and Trinidad and Tobago and the southern offshore islands because they are south of the common hurricane boundary. The analysis is supplemented with a number of incomplete domestic structures from other sites and regions by virtue of the shared building choices apparent in their archaeological plans.

Table 1 presents 16 sites across the Caribbean for which ca. 150 structures have been published. Individual sites frequently demonstrate a palimpsest of multiple centuries of habitation, although the lack of radiocarbon dates from house features provides little intra-site chronological control. Structures date from 1400BP to 450BP, or from the start of the Late Ceramic Age expansion up to European colonization, with most evidence available from 1100BP onwards. Around 80 of these structures have been interpreted as houses (as opposed to mortuary, ancillary or boundary structures). Site types range from isolated structures perhaps representing seasonal or garden houses, such as Playa Blanca 5, multiple households such as Kelbey’s Ridge 2, to large settlements, or towns,^3^ such as El Cabo and Tutu. The number of structures per site is a reflection of the size of the excavated area rather than the settlement size. Not all sites yielded equally reliable plans nor all publications sufficient detail, and the discussions below rely to a certain extent on interpretive bootstrapping from data from sites such as El Cabo, Tutu and Anse à la Gourde for which multiple structures are available (Hofman *et al*. 2001; Righter 2002; Samson 2010, 2013; Bright 2003; Morsink 2006).

**Table 1:** Overview of structures discussed. Period refers where possible to date ranges for houses, and otherwise site dates. Only the most reliable/complete of these structures have been used in analysis and where multiple interpretations have been presented the most secure and parsimonious are included

	Site name	Period BP	No. houseplans	Construction	Area (m^2^)	Environmental setting	References
Cuba	Los Buchillones	655–260	3 (of al least 5)	Stilted (?), post-build, circular and rectangular	45 to 530	Coastal wetland, caves close proximity	Jardines Macías and Calvera Rosés 1999; Pendergast et al. 2002; 2003; Valcárcel Rojas 2005, 2006
	Loma del Convento	650–450	2	Post-built, circular and rectangular	13	River valley hilltop, <4 km from coast	Knight 2010
Turks and Caicos	MC-6	550–450	8	Round pit structures in raised lime-stone bank	20	Coastal, tidal flat. Low rainfall, high winds	Sullivan 1981 in Keegan 2007; Keegan et al. 2008
Jamaica	Bellevue Mannings Hill	1050–450	1	Post-built, circular	10	In hills, 8 km from coast	Medhurst 1976, 1977; Allsworth-Jones 2008
Dominican Republic	El Cabo	1050–450	30	Post-built, circular	19 to 100	Coastal, 5 m asl. Caves close proximity	Samson 2010
Puerto Rico	Maisabel	1350–750	1 (and up to 3)	Post-built, rectangular	576	Atlantic coast	Siegel 1989, 1992; Curet 1992
	El Bronce	1050–750 and 750–450	(at least) 3	Post-built, oval and circular	20 to 24	13 km from coast	Robinson *et al*. 1983, 1985; Curet 1992
	Lujan I	1050–750	8 (10 inc. mortuary structures)	Post-built, circular	13 to 346	Hilltop, 3 km from coast	Rivera and Pérez 1997
	Rio Tamaná	970–460	7	Post-built, oval and circular	20 to 50	Alluvial plain, 8 km from Atlantic coast	Carlson 2007
	Playa Blanca 5	750–450	1	Post-built, circular-oval	37 or 200	Wetland hilltop, overlooking sea < 2 km	Rivera and Rodríguez 1991; Curet 1992
	Rio Cocal-1	1060–500	4 or more	Post-built, circular	10 to 24	Atlantic coastal plain, caves close proximity	Goodwin *et al*. eds., 2003, Oliver 2003
US. Virgin Islands	Tutu	1885–1000 and 800–450	8	Post-built, oval and circular	12 to 19	River valley; 1.75 km from coast	Righter 2002
Saba	Kelbey’s Ridge 2	650–600	5	Post-built, circular	57 to 80	Ridge 140 m asl, 300 m from coast	Hoogland 1996
St. Eustatius	Golden Rock	1350–1050	6	Post-built, circular	41 to 283	Centre of island, < 1.5 km from coast	Schinkel 1992
Guadeloupe	Anse ála Gourde	970–520	13 (of ca.24)	Post-built, oval and circualr	27 to 130	Atlantic coast	Bright 2003; Duin 1998; Hofman et al. 2001; Morsink 2006
	La Pointe de Grande Anse	1350–1150	1 (and up to 4)	Post-built, circular-oval	165 (from paln)	River bank, on coast	Van den Bel and Romon 2010

In terms of settlement location all sites are situated on the shore or are within several kilometres of the coast (max. 13 km), often on elevated landscape features such as hilltops or artificial mounds. In general settlements are positioned to exploit diverse terrestrial and marine resources and access to shelter, often sited near coral reefs and caves. Those settlements on the Atlantic coasts are more exposed than those on the Caribbean coast, and those on larger islands, or those on islands with greater elevations experience more precipitation than lower, smaller islands.

## The Houses of El Cabo

The indigenous town site of El Cabo in the eastern Dominican Republic exemplifies many of these characteristics. The extensive excavations at the site have doubled the number of indigenous structures known from the Greater Antilles. Researchers from the Museo del Hombre Dominicano first investigated the El Cabo site in the late 1970s, and again between 2005 and 2008 in partnership with Leiden University.^4^ The goal of the research in El Cabo was to develop an archaeological perspective on the indigenous house and settlement dynamics (Hofman *et al*. 2006, 2008; Samson and Hoogland 2007; Samson 2010, 2011). These aims were pursued by a horizontally extensive fieldwork strategy that documented habitation features, domestic structures and settlement space.

**Fig. 3:**
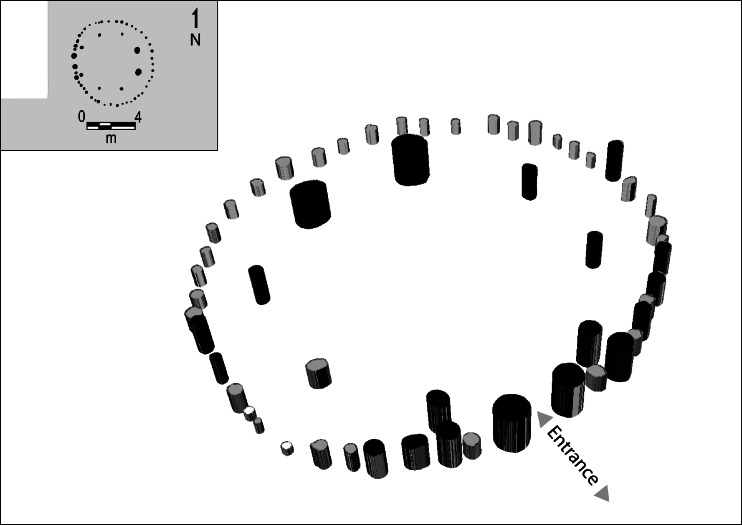
3D rendition of the plan of house Structure 1, El Cabo, Dominican Republic. Note the internal symmetries of the plan: the two close-set entrance postholes align on an internal configuration of eight paired posts, probably supporting tie-beams and a ring beam. The entrance is flanked by alternating larger and smaller postholes of decreasing size, with smaller postholes in the back of the house. The prevailing easterly wind would have been channelled over the roof creating a sheltered outdoor space in front of the house

El Cabo is situated on a coastal promontory surrounded by coastal caves a kilometre inland and overlooking the Mona Passage to Puerto Rico, waves breaking over a coral reef a short distance offshore. Excavation of the main unit (1000 m^2^) revealed over 2000 archaeological features. The overwhelming majority of these features are postholes, the unique preservation of which, directly cut into the limestone bedrock, enabled identification of over 50 structures, 30 of which are houses, in addition to a platform structure, storerooms for community regalia, fences or palisades, windbreaks and work huts. From 1100BP to around a decade after European colonization El Cabo was a town consisting of some half a dozen neighbouring groups of houses arranged along the coast. Individual houses were periodically rebuilt, or renewed. The archaeological traces of this process are sequences of multiple, contiguous, overlapping house-plans, or House Trajectories (Samson 2010: fig.152). Individual house structures were circular, between 6.5 and 10 m in diameter, and consisted of an outer perimeter wall of closely-spaced, slender posts, and eight, larger, roof-bearing posts, aligned on an inland facing doorway (Fig. 3). House structures (n = 31) share specific architectural features, are consistently larger and more symmetrical in plan than other structures (n = 21), are associated with certain types of artefacts and ritual practices, and were rebuilt multiple times.

The archaeological evidence attests to the multifunctional character of the house, in which domestic spaces were also social and political institutions and the location of daily activities and long-term community investments. Accumulated debris around the houses indicate the preparation and consumption of meals, pottery for cooking and serving, and tools for subsistence and craft activities; ritual objects indicate the consultation of ancestors and community ceremonies; human remains indicate mortuary rituals; and the placing of personal effects in postholes on the abandonment of the house indicate its intimate relationship with the lives of its inhabitants.

The high-resolution archaeological data from El Cabo enable an expansion of the geographic scale of analysis and a re-evaluation of structures from other sites with fewer houses and for which physical data or detailed plans are lacking. The institutional durability of some of these houses, apparently over millennial timescales, is witnessed in the house trajectories, which form long-lived estates whose members were likely able to trace their ancestors back to common origins (Samson 2010).

## The Caribbean Architectural Mode

The plans from the sites in Table 1 belong to small, round and semi-round post built structures. Many have internal features such as hearths and in some human burials were found under the house floors. Features exterior to the structures include pathways, fences, and small ancillary buildings, interpreted as kitchens, windbreaks, and mortuary structures. Although a systematic study of these features and associated artefact assemblages would be an important step in defining whether the structures fulfilled similar roles, this is beyond the scope of the current paper. In addition to similarities in settlement location at least seven shared construction characteristics can be identified which are shared across sites and islands:

### Architectural Footprint

Firstly, the majority of structures are circular to oval and use a combination of large and small posts distributed regularly throughout the building. There is a clear distinction between heavier, roof-bearing posts, and smaller wall elements. Deep foundations are a recurrent feature of Caribbean architecture (Mason 1941; Hofman *et al*. 2012; Schinkel 1992), although the hardness and durability of tropical hardwoods, such as mahogany and sapodilla, mean that even slender posts could have supported considerable loads. For example, 90 % of postholes in El Cabo are less than 26 cm across, and over 25 % are between 12 and 14 cm in diameter, which means the posts themselves were even slighter. Postholes in El Cabo fall into diameter classes that likely correspond to the most common dimensions for construction timbers, suggesting standardization and availability of suitable trees. The same may not have been the case for smaller islands such as the Turks and Caicos where high winds and lack of rainfall may have limited availability of suitable timbers. This is indicated by structures on MC-6, which rely on stone construction material probably in addition to timber. Walls of woven vines (*bejucos*), or open walls would have allowed breeze to circulate inside structures, with the use of windbreaks to mitigate strong gusts and provide sheltered areas to work in and around houses (evidence for which is found in Tutu, Golden Rock, El Cabo, and Anse à la Gourde).

European roundhouse studies indicate that structures up to 12 m in diameter have no need of internal roof supports (Pope 2008). Nevertheless, even though most of the Caribbean structures are considerably smaller, numerous solutions are adopted for supporting the roof: by the external wall, internal post rings or sometimes a central post (s). Structures from several sites in Puerto Rico share the same heavier four-post framework incorporated into the outer wall - a pattern first remarked upon by Rivera and Rodríguez (1991) for Playa Blanca 5 (Curet 1992) and occurring more frequently at El Bronce and Río Cocal-1. Four-post central configurations occur at several sites across the Caribbean at Río Tanamá (Structure 6), Luján I and Tutu (Structures 2 and 7), Anse à la Gourde (multiple structures), La Pointe de Grande Anse (locations 1 and 2) and Kelbey’s Ridge 2 (structures 1 to 4). Elsewhere, such as Los Buchillones and El Cabo, inner post rings provide roof support. A centre post (s) is not standard, occurring only in some structures in less than half of sites.

### Size

**Fig. 4:**
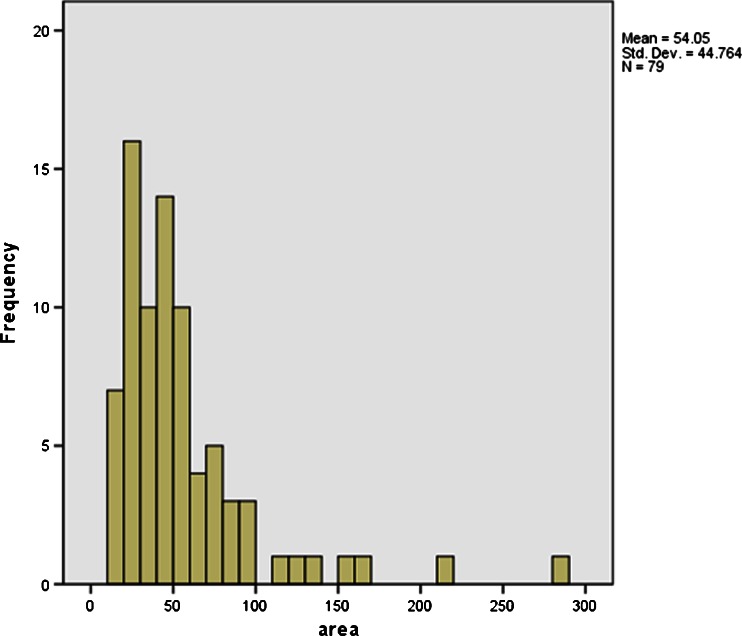
Histogram showing the frequency of house floor areas. This table excludes five larger structures from Cuba (Los Buchillones, 530 m^2^), Puerto Rico (Maisabel, 576 m^2^, Luján I, 346 m^2^) and Haiti (En Bas Saline, two oval plans 15 m diameter) due to their incompleteness or lack of published details

Overall, houses are small structures, which fall most credibly within the range of 20 to 60 m^2^, with an average area of 54 m^2^ (Fig. 4). Using Antonio Curet’s formula for estimating prehistoric populations (1998) this equates to four to eleven inhabitants per structure, which may represent a small extended family, or imply the distribution of members of an extended household over multiple structures.

In terms of chronological variation, there are currently neither enough radiocarbon dates nor a body of reliable early plans to identify trends. A reported decrease in house size over time for eastern Puerto Rico, referred to earlier, needs to be tested with more comparative, regional datasets (Curet 1992; Curet and Oliver 1998). In terms of geographic distribution, size variation occurs within and between sites. Houses in the Greater Antilles are on average smaller than in the Lesser Antilles. The largest size category contains several structures from sites in the northern Lesser Antilles including the three maloca-style houses from Golden Rock. In general, houses in all periods in the study region are considerably smaller than ethnographic examples from the South American mainland and archaeological examples from the southern Caribbean islands and mainland (Schinkel 1992:184; Oliver 1995; Versteeg and Rostain 1997).

### High-Pitched Roofs

Thirdly, houses probably had high-pitched roofs. This is suggested for Tutu, Playa Blanca 5, Golden Rock and El Cabo. Archaeological evidence from El Cabo indicates the roof-pitch for Type 1 houses was 40° based on the incline of the postholes of the outside wall, which also formed the roof (Samson 2010:239). At Golden Rock, Schinkel calculates roof pitch as a steep 0.8 × diameter (1992: 192).

### Monumental Facades

**Fig. 5:**
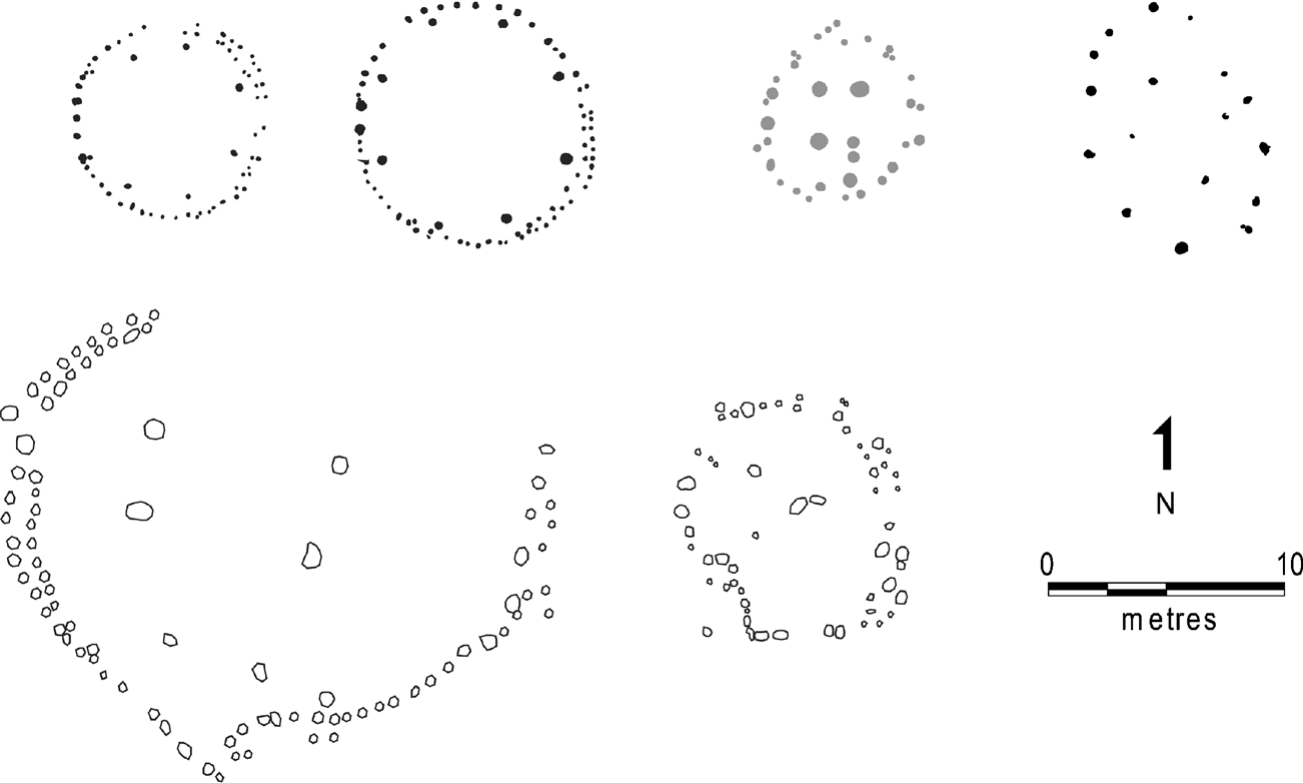
House plans from top left to bottom right: El Cabo (structures 4 and 14, *black*), Tutu (structure 2, *grey*), Anse à la Gourde (structure 2, *black*) and Luján (structures 1 and 6, *white*)

Fourth, house facades may have been reinforced and emphasized. This is most apparent in the case of El Cabo where large posts either side of the entrance run up to a third of the perimeter of the house, becoming smaller and shallower towards the back (Fig. 3). All house structures in El Cabo and three from Tutu (Structures 1, 2 and 6) have either double or enlarged entrance features. This can be seen in Fig. 5, which shows two structures from El Cabo with characteristic narrow, but monumentalized westerly entrances, and a structure from Tutu with an east-facing portico represented by doubled postholes (Righter 2002:316). Although not explicitly stated by excavators, the plans indicate that structures at the sites of Luján I (Structures 1, 6 and partial 7) and Río Tanamá (Structure 2) may have had doorways consisting of paired, heavy-set posts. In the case of Luján I these entrance features appear to align on internal configurations, as in El Cabo, and open onto a central clearing (Rivera and Pérez 1997).^5^ In general doorways are narrow and low, admitting one person at a time and perhaps requiring the individual to duck. It is not clear whether the structures from the Lesser Antilles share this feature.

### Prepared Floors

Fifth, there is evidence for prepared floors. In some sites creating a level living surface was relatively easy, whereas in others it required significant effort. At Playa Blanca, for example, an irregular surface was cleared and levelled by removing rubble and rocks, and in Kelbey’s Ridge 2 the gravel of the house floors had been compacted. In El Cabo, rather than concentrating building activities in areas of softer, sandy deposits, the hard, uneven peaks of the limestone had been removed to form a flat surface inside structures.

### Securely Anchored Foundations

**Fig. 6:**
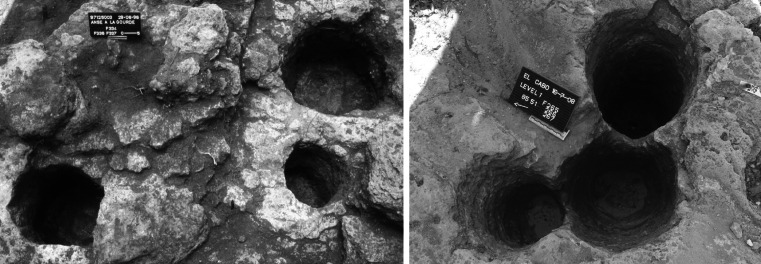
Postholes cut into the bedrock in Anse à la Gourde, Guadeloupe (*left*), and El Cabo, Dominican Republic (*right*). Note the regularity of the features

Sixth, there is a preference across the region for selecting the bedrock for securing house foundations (Table 2). Where this is the case, either a select number of the larger posts are anchored in the limestone or volcanic bedrock, such as at Anse à La Gourde and Golden Rock, or the majority of the postholes are bored directly into the bedrock. This is the case in El Cabo where postholes ranging from a few centimetres to well over a metre deep are hewn into hard limestone. These features are extremely regular in plan and cross-section, probably closely correlated to timber dimensions, and must have been made with great skill using perhaps shell picks or chisels (Fig. 6). An alternative means of anchoring structures is seen at MC-6 on Middle Caicos where stone-lined, semi-pit circular structures were set in raised limestone banks (Keegan 2007).

**Table 2:** Sites with postholes cut into the bedrock

	Sites	References
Cuba	Loma del Convento	Knight 2010
	El Morillo	Hernández and Rodríguez 2008
Dominican Republic	El Cabo	Samson 2010
	Macao	Andújar Persinal *et al*. 2004:171
	Caletón Blanco	Olsen Bogaert 2001, 2002
	Soco	pers. comm. Veloz Maggiolo
Puerto Rico	Playa Blanca 5	Rivera and Rodríguez 1991
	Luján I	Rivera and Pérez 1997
St. Eustatius	Golden Rock	Schinkel 1992
Guadeloupe	Anse á la Gourde	Bright 2003; Duin 1998; Hofman *et al*. 2001; Morsink 2006
	La Pointe de Grande Anse	Van den Bel and Romon 2010
Florida, U.S.A.	Miami Circle	Wheeler and Carr 2004
	Palm Royal Circle	Collins *et al*. 2006; Wheeler and Carr 2004

Despite the fact that there is currently little evidence for regular contact between the south eastern United States and the Caribbean islands in prehistory, we nevertheless include two sites from what is today downtown Miami in this discussion because of the striking similarities in terms of the physical characteristics and technological choices (Wheeler and Carr 2004; Collins *et al*. 2006). Although ostensibly dating to over 500 years earlier than the Caribbean houses, the coastal setting of the sites and their overlapping, concentric and regular circular structures in the upper size range of the Caribbean house floors (106 m^2^), with postholes cut into limestone bedrock, are quite at home in the tradition of the bedrock architecture of the islands.

### Durability: Repair and Rebuilding

Lastly, it appears many structures endured a considerable length of time through either rebuilding or the replacement of parts. This characteristic longevity was already observed for settlements in the northern Lesser Antilles by Versteeg et al. (1993) and Schinkel ( 1992) and contrasted with shorter-lived villages in the South American lowlands.

Figure 5 shows rebuilding and repair in the doubling of features of the perimeter walls of structures in El Cabo, and we suggest, along the back walls in Luján I. Replacement of structural elements and evidence for repair can be seen at most sites. One of the most striking examples of longevity through repair is house 1 from Los Buchillones, Cuba, with a lifespan of 360 years (Pendergast *et al*. 2002).

**Fig. 7:**
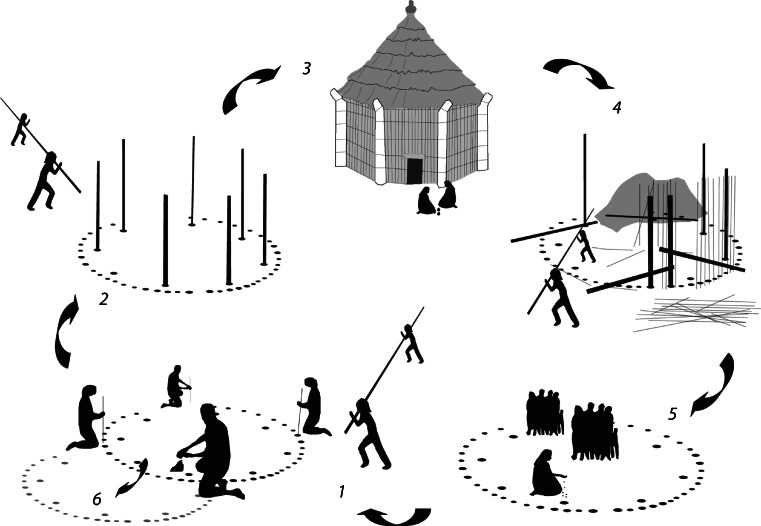
Repair and rebuilding in the lifecycle of a house. *1*) digging foundations, *2*) construction, *3*) habitation & cycles of repair, *4*) abandonment and re-use of parts, *5*) ritual closing, *6*) starting anew. Based on Samson 2010; fig 155

Site occupation typically spans several centuries with the number and spatial distribution of features often relating to two and up to five related building phases. This is indicated by the feature clustering at sites such as Tanamá, Luján I, Maisabel, Río Cocal-1, El Bronce, Tutu, Golden Rock, Anse à la Gourde and La Pointe de Grande Anse, and particularly exemplified by the house trajectories in Kelbey’s Ridge 2 and El Cabo with an eleventh century date for one of the first houses, and fifteenth century colonial material directly related to the last house in a related sequence at the latter site.

These data from many house plans and sites show that common house features appear in the archaeological record after 1400BP. The emergence of this mode coincides with widespread transformations in indigenous society of which these changes were a part. By 1100BP a process of house definition and elaboration was well underway which enhanced people’s capacity to carry out daily tasks, gain political and institutional leverage, transmit memory, and deal with the environment, similar to the role of the house in non-industrial societies worldwide (Beck ed. 2007; Joyce and Gillespie 2000). The house both constituted changes and catalysed opportunities to make things possible.

## Discussion

### Geographic Scales and Timeframes of Interest: Modes Shared by People Across a Region and Over Time

These regional data show that indigenous communities across the Caribbean, and arguably further afield, shared and developed house-building strategies comprising small, carefully designed and evenly anchored structures with high-pitched roofs and reinforced facades. These common features emerged in areas with similar ecologies and were well adapted for dealing with the winds, precipitation and heat of the Caribbean. Settlement characteristics such as the irregular layout of houses and the use of windbreaks and partitions reduced exposure to wind. Lastly houses experienced centuries of longevity due to deliberate prolongation of their lives through rebuilding and repair.

### Perspectives on Change: Rates and Means of Repairing and Rebuilding

These long-range data suggest that houses would have performed well both surviving an event and in speed of reconstruction (supporting Cooper and Peros 2010). The foundations were secure in high winds and earth tremors, in part because long, dense timbers are heavy enough to resist uplift.^6^ Making postholes in the bedrock would have facilitated house dismantlement at the approach of extreme events (the main posts could be laid flat); kept intact the most valuable and labour-intensive parts of the construction (large support posts and foundations); and allowed rapid repair and reuse after initial storm impact (posts could be reassembled in the original foundations). The same postholes may have been reused multiple times for multiple replacements of the same house (Fig. 7). Moreover, as has been noted for Los Buchillones, the shedding of building materials would cause relatively little harm to inhabitants (Cooper and Peros 2010). Ease and speed of dismantlement may also have favoured smaller rather than larger houses, and the choice of smaller, and thus more numerous dwelling structures may have increased building survivorship ratios. Houses thus incorporated and shared a “sacrificial principle” by virtue of their combination of robust and replaceable lightweight elements providing an effective recovery system. It is this technology which European colonizers misinterpreted as expedient and insubstantial.

The means of prolonging house life - repair, dismantlement, reusable post-holes and replaceable elements - do not in themselves explain why house trajectories endured. Instead, these data show frequent, cyclic adaptations to the weather, infrequent fundamental change over time, and when change does occur, it is for multiple complex reasons. The mode persisted, in the terminology of McGlade (1995), even as instabilities and tensions were playing out at generational, seasonal and daily timescales, exhibiting its ability to both absorb and utilise change at different scales, from climatic variability to periodic storms, and the waxing and waning of family groups to political upheavals and wider social change. A theory of change, on this evidence, must consider the house as both social process and (technological) product in which no pathway predictably arrives at a stable, fully explicable or permanent house.

### Treatment of the Evidence: Unknowable Motives for Repairing and Rebuilding

The data confirm one mode’s effectiveness at mediating the environment: evidence for other modes and other factors influencing building choices is beyond the scope of this paper. Even within the mode, the variation between sites shows there was no natural template, rather the mode’s characteristics were contingent upon and encoded in diverse ecological and non-ecological relations. For example, house form expressed cosmological principles (Siegel 2010); house size corresponded to patterns of household mobility and demography (Curet 1992; Veloz Maggiolo 1984); settlement location was a trade-off between proximity to neighbours and resources, and exposure to climate risk (Keegan *et al*. 2008; Cooper and Peros 2010); roof height and elaborate facades played a role in exhibition (Pané 1999) and signified the identities and affinities of house members; and digging foundations, the selection of materials, and house construction structured community lifecycles. In El Cabo, despite the fact that postholes in the bedrock offered the possibility of infinite re-use, inhabitants periodically built new foundations, possibly as part of coordinated periods of community renewal - a choice that cannot be reduced to functionalist explanations.

The archaeological data presented here offer a time-depth lacking in contemporary humanitarian responses, complement studies of past human-environment relations by focusing on individual structures, and examine house building technologies that are shaped both by maritime ecologies and by shared cultural practices that made the house central to community identity and practice.

The emergence, formalization and persistence of this particular Caribbean building mode experienced less frequent fundamental change over time than humanitarian conceptualisations of shelter might predict, and instead exhibits certain useful features and more cyclic adaptation to the weather.

Ultimately, we hope that considering this long-term, regional analysis of house features might contribute to greater engagement between archaeologists and those responsible for building (or rebuilding) the present.

## Footnotes

1 http://www.ifrc.org/en/what-we-do/disaster-management/responding/services-for-the-disaster-affected/shelter-and-settlement/how-we-do-shelter/

2 Carlson 2007; Curet 1992; Delpuech *et al*. 1997; Drewett and Bennell 2000; Goodwin *et al*. eds. 2003; Hofman *et al*. 2012; Hofman and Hoogland 1999; Hofman and Hoogland 2010; Hoogland and Hofman 1993; Hoogland 1996; Jansen and Dorst 2007; Jardines Macías and Calvera Rosés 1999; Kaplan 2009; Meléndez Maíz 1996; Pendergast *et al*. 2002, 2003; Righter 2002; Rivera and Pérez 1997; Rivera and Rodríguez 1991; Schinkel 1992; Siegel 1989, 1992; Sullivan 1981 in Keegan 2007; Rojas *et al*. 2006; Van den Bel and Romon 2010; Versteeg and Rostain eds. 1997; Walker 2005.

3 The term “town” is used to express comparative population size with settlements in Europe. Indigenous towns were socially, economically and administratively distinct from Old World models.

4 Directed by Dr Menno Hoogland, “Houses for the living and the dead, organisation of settlement space and residence rules among the Taino, the indigenous people of the Caribbean encountered by Columbus”. The Netherlands Organisation for Scientific Research, project nr. 360-62-030.

5 Large posthole pairs in the southeastern perimeter of Luján Structure 1 and the western perimeter of Structure 6 can be discerned in Fig. 5; however, without publication of further details this remains tentative.

6 Uplift is the vertical upward force from wind lifting out foundations – this is a result of the suction effect of a lower wind pressure on the leeward face of a shelter or roof surface. Transitional Shelters supplied in Haiti after the 2010 earthquake (rectangular 5 m × 4 m in plan) were designed with blocks of 0.2 to 0.3 cubic metres of concrete as foundations that would weigh enough to resist uplift. For a Category 1 hurricane (108 mph or 43 m/s gust speeds), calculated uplift forces on these shelters varied between 4kN (corners) 7kN (middle post). Depending on the height, strength of connections and permeability of the walls and roof of the El Cabo houses, uplift would be of a similar order and mahogany posts (density of 660 kg/m3 or about a third of concrete) with the diameters and depths recorded would indeed resist uplift forces of 3 to 4kN at the monumental façade and an average of 1.75 for the other 52 posts.
